# Effectiveness of therapeutic hypothermia for mild neonatal encephalopathy: A protocol for systematic review and meta-analysis

**DOI:** 10.1097/MD.0000000000029175

**Published:** 2022-05-27

**Authors:** Tingting Zheng, Xini Liu, Xuechun Chen

**Affiliations:** a Department of Pediatrics, the First Affiliated Hospital of Hainan Medical College, Hainan, China; b Department of Pediatric Emergency, the First Affiliated Hospital of Hainan Medical College, Hainan, China; c Department of Respiratory Medicine, Union Jiangbei Hospital of Huazhong University of Science and Technology, Hubei, China.

**Keywords:** meta-analysis, neonatal encephalopathy, systematic review, therapeutic hypothermia

## Abstract

**Background:**

Neonates with moderate to severe encephalopathy benefit significantly from therapeutic hypothermia, with reduced risk of death or disability. However, the need for therapeutic hypothermia for mild neonatal encephalopathy (NE) remains unclear. Therefore, we conducted a protocol for systematic review and meta-analysis to provide evidence supporting therapeutic hypothermia for term or near term neonates with mild NE, including findings of recent long-term outcome studies, as well as novel adjunctive therapies to augment neurodevelopmental outcomes for neonates with NE who receive therapeutic hypothermia.

**Methods:**

Two independent researchers performed a systematic literature search in different electronic databases including PubMed, the Cochrane Center Controlled Trials Register, EMBASE, Medline, Ovid, Chinese National Knowledge Infrastructure, Chinese Biomedical Literature Database, and Wanfang Database without any restrictions of languages and date. Two reviewers will screen the records and include quality studies according to inclusion criteria independently. Two reviewers will assess the risk of bias of the included studies by the “Risk of Bias Assessment Tool” of the Cochrane Handbook for randomized controlled trials. Statistical analysis will be performed with Review Manager software 5.3.

**Results:**

A synthesis of current evidence of therapeutic hypothermia for treating mild NE will be provided in this protocol.

**Conclusion:**

The results of this study will provide a theoretical basis for the clinical use of therapeutic hypothermia in mild NE.

## 1 Introduction

Neonatal encephalopathy (NE) is identified by the World Health Organization as one of the 10 leading causes of lost years of life and is extremely expensive in lifetime costs and in emotional distress for individuals and families.^[1]^ Although NE is traditionally attributed to birth asphyxia, population-based studies have consistently observed that most NE and some of the subset of NE considered to be hypoxic-ischemic in origin occurs in infants who have not experienced acute peripartum or intrapartum events.^[2]^ NE was characterized by a subnormal level of consciousness, depressed tone, and reflexes, with or without seizures and often with impaired respiration and feeding abilities (both of presumed central origin).^[3–5]^ It is defined as mild, moderate, or severe based on the Sarnat scoring system.^[6]^

Neonates with mild NE have not been included in previous trials of therapeutic interventions because they were previously thought to have neurodevelopmental outcomes similar to those without evidence of NE.^[7]^ In the therapeutic hypothermia era, reviews have found that 20% to 40% of neonates with mild NE (varying definitions) have abnormal short and long-term outcomes.^[8,9]^ In clinical practice, providers are becoming increasingly concerned about neuroprotection for neonates with mild NE, as there is a trend towards providing therapeutic hypothermia to these babies without clinical evidence of benefit.^[10]^ Neonates with mild NE initially appear well with few overt signs of neurological injury. However, the effects of therapeutic hypothermia for mild NE have never been systematically evaluated. Therefore, we conducted a protocol for systematic review and meta-analysis to provide evidence supporting therapeutic hypothermia for term or near term neonates with NE, including findings of recent long-term outcome studies, as well as novel adjunctive therapies to augment neurodevelopmental outcomes for neonates with NE who receive therapeutic hypothermia.

## 2 Methods

### 2.1 Study registration

This systematic review has been registered on Open Science Framework (Registration number: 10.17605/OSF.IO/GA5HK) and will be performed according to the Preferred Reporting Items for Systematic Reviews and Meta-Analysis Protocol statement guidelines.^[11]^ Ethical approval was not required for this study as all the research materials are derived from published studies.

### 2.2 Selection criteria

Randomized controlled trials (RCTs) comparing selective head or whole-body cooling with usual care in term or near-term infants (≥36 weeks) with mild NE after perinatal asphyxia were eligible. Perinatal asphyxia required at least one of the following criteria: evidence of intra-partum catastrophe, fetal or neonatal metabolic acidosis, and/or resuscitation at birth. Mild NE was not defined separately but was based on a clinical neurological examination performed within 6 hours of birth, as reported in the individual studies. Main outcomes were the composite of death or moderate or severe disability at or beyond 18 months of age. The exclusion criteria were as follows: reviews, letters, conferences abstract, case reports or series, comments, and animal experiment.

### 2.3 Literature search

Two independent researchers performed a systematic literature search in different electronic databases including PubMed, the Cochrane Center Controlled Trials Register, EMBASE, Medline, Ovid, Chinese National Knowledge Infrastructure, Chinese Biomedical Literature Database, and Wanfang Database without any restrictions of languages and date. Search only Chinese and English literature. The database search was carried out in the form of subject headings combined with free words. The search terms included “hypoxic ischemic encephalopathy,” “neonates,” and “hypothermia.” In addition, references to the included literature were traced back to supplement the acquisition of relevant literature.

### 2.4 Selection of studies

Two reviewers will independently review the titles, abstracts, and full text of the studies for eligibility for inclusion in this systematic review. All studies searched by electronic databases and identified by hand will be organized in Endnote X7 (Thomson Reuters, New York, NY). Any disagreement between the 2 reviewers will be resolved by discussion and consensus. If necessary, a third reviewer will intervene and resolve any disagreement.

### 2.5 Data extraction

Two review authors will independently extract the data and fill out the standard data extraction form, which includes study information such as the first author, publication year, title, journal name, research design, number of patients, inclusion criteria, interventions, control, treatment period, outcome measures, and adverse events. Data extraction will be performed by 2 independent investigators according to a predesigned review form. Disagreements are resolved through discussion among all authors.

### 2.6 Risk of bias assessment

Two reviewers will assess the risk of bias of the included studies by the “Risk of Bias Assessment Tool” of the Cochrane Handbook for RCTs.^[12]^ The evaluation contents include random sequence generation, allocation concealment, blinding of participants and personnel, blinding of outcome assessment, incomplete outcome data, selective reporting, and other biases. Each item is divided into “high risk,” “unclear risk,” and “low risk.” Any inconsistencies will be determined in consultation with the third reviewer.

### 2.7 Data synthesis and analysis

Statistical analysis will be performed with Review Manager software 5.3 (Cochrane Collaboration, Nordic Cochrane Center, Copenhagen, Denmark). Relative risk is used to evaluate the effect size for binary variables, and the mean difference is used as the efficacy analysis statistic for continuous variables. Heterogeneity between results will be assessed by the value of *P* and *I*^2^. When *P* > .1, *I*^2^ < 50%, it will be considered as no significant heterogeneity between the trials, and the fixed-effect model will be applied for statistics, otherwise, the random-effect model will be chosen.

### 2.8 Sensitivity analysis

If the risk of bias of the studies is high, sensitivity analysis will be performed to investigate the asymmetry of funnel plots to exclude low-quality studies.

### 2.9 Assessment of quality of evidence

We will use the Grading of Recommendations Assessment, Development, and Evaluation to assess the results.^[13]^ In the Grading of Recommendations Assessment, Development, and Evaluation system, the quality of evidence will be categorized into 4 levels: high, moderate, low, and very low quality.

## 3 Discussion

Evidence from RCTs has confirmed that therapeutic hypothermia to neonates with moderate to severe NE resulted into reduction in mortality and major neurodevelopmental disability at 18 months of age.^[14]^ As a result, therapeutic hypothermia is currently the mainstay of treatment in neonates with moderate or severe NE.^[15]^ Neonates with mild NE were not part of the eligibility criteria in the trials of therapeutic hypothermia as they were previously considered to have good long-term outcomes.

Recently, evidence is emerging that even mild NE can adversely affect long-term neurodevelopmental outcomes. Studies have suggested that without the application of therapeutic hypothermia, neonates with mild NE have equal risk of having brain magnetic resonance imaging abnormality (54% in mild vs 54% in moderate encephalopathy) and disability as compared with the infants with moderate encephalopathy at 5 years of age.^[16]^ However, the beneficial effects of therapeutic hypothermia in mild NE must be proven in high quality studies before it could be used as a standard of care. To the best of our knowledge, this is the first meta-analysis of RCTs to evaluate the effectiveness of therapeutic hypothermia for mild NE. These clues and conclusion are hoped to encourage researchers to conduct further research on the subject.

## Author contributions

**Conceptualization:** Xini Liu.

**Data curation:** Tingting Zheng, Xini Liu.

**Funding acquisition:** Xini Liu, Xuechun Chen.

**Investigation:** Tingting Zheng.

**Methodology:** Xini Liu.

**Writing – original draft:** Tingting Zheng.

**Writing – review & editing:** Xuechun Chen.
